# Practical Departments

**Published:** 1904-12-31

**Authors:** 


### PRACTICAL DEPARTMENTS.

### MILK POWDERS.

SOME few months ago attention was drawn in these columns to the fact that large hospitals, with their power of purchasing meat advantageously in the open market, could make for themselves cheaper and more nutritious beef-tea than they could obtain by the use of meat extracts which certain companies with dividends to earn were eagerly—and in some cases apparently successfully—pressing on their acceptance.

It would seem that hospital officials are supposed to be unusually gullible, for recently attempts have been made to induce them to use certain dried milk powders in preference to purchasing fresh milk. It has been claimed that these milk powders are far superior to ordinary milk, that they are free from all bacteria, so that sterilisation is unnecessary, and that they are highly nutritious, containing in proper proportions all the ingredients necessary for the nourishment of the body.

Since one of these wonderful preparations was offered at the same price as a large hospital paid for its fresh milk, one naturally asked the question, "How is it that the manufacturer can afford to sell it so cheaply?" He can scarcely get his raw material cheaper than a large institution, presuming he takes as much trouble to get a pure and genuine milk. He then has to submit it to a manufacturing process, to pack it, to advertise it, and to sell it at a profit. Why then is it not dearer than ordinary milk? Analysis revealed the reason and showed that the article was no substitute for fresh milk. The milk fat was but 1·25 per cent., that is to say, the powder was deficient in cream to the extent of no less than 60 per cent.

On this fact being notified to the representative, who was clamouring for orders, the colossal ignorance of some of the persons who are engaged in this business was amusingly manifested. "Oh!" said he, "that is all right, we have here a powder we can send you containing 27 per cent. of cream!" This, in face of the fact that the Government standard is 3 per cent., and that even a Jersey cow can only give about 5·6 per cent.

The claim that the powder is germ free would seem to be equally baseless, for, whilst the method of packing does not preserve it from contamination by the air, the process of reconstitution with hot water necessarily creates a liquid that is at a particularly favourable temperature for the propagation of bacteria. Another powder which was submitted to examination, after reconstitution in accordance with the manufacturer's directions, was found to contain 3·15 per cent. of fat but only 11·11 of total solids—that is to say, it was equal to a milk with 6 per cent. of added water.

In view of these facts it would seem important that hospitals should be careful to ascertain for themselves the precise constituents of any milk powders before deciding to use them.

### THE STOLZENBERG FILE SYSTEM.

The Stolzenberg system of filing is applicable to all classes of clerical work, and it will be found equally useful in the secretary and matron's office, the writing-table of the medical man, the business house, or in the hands of all methodical members of the community. One specially useful feature of the files is that they are made in different colours, which greatly facilitates speedy reference. They are simple, strong, and inexpensive, three good qualities which should be sufficient to recommend them. All the directions in which the system is applicable, and its numerous modifications, are indicated clearly in the descriptive and illustrative catalogue of the Stolzenberg Company, Leonard House, 50 Bishopsgate Without. The system which serves to bind manuscript music, newspapers, and literary matter of every kind, is so comprehensive that we recommend our readers to seek in the pages of the catalogue for any description of file they may need to assist them in their particular occupation, and we predict with confidence that they will find it. A personal experience of the use of the Stolzenberg files has rendered them practically indispensable.

### IMPROVEMENTS AT THE CHELSEA HOSPITAL FOR WOMEN.

The Chelsea Hospital for Women has lately undergone some important alterations in its operating theatre and sterilising room in order to render these departments as perfect as possible. One marked improvement is the conversion of the old sterilising room into the theatre anteroom. The new sterilising room, though on the same floor, is far enough away to prevent any heat from the boilers being felt in the theatre, and the sterilised water is conveyed into the theatre by pipes. The ante-room is fitted with washing basins for the surgeons and nurses. In the theatre itself a special new feature is a mirror, for concentrating light on the operating table, fitted with four electric lamps, supplied by Wright Bros. The lower part of the visitors' gallery has been taken away to make room for this mirror. Other innovations are some surgeon's arm lotion basins, by Allen and Hanbury, an anæsthetist's glass and white enamel table by Down Bros., and a new washing sink, with slab, for nurses. All the washing basins and sinks are worked by pedal movements instead of hand water-taps, and for these fittings Messrs. Davies and Bennett are responsible. The electric lights too are all switched on by foot. Altogether the theatre as it is now is extremely well equipped, some of the fittings being exceptionally good.

